# Nominating Committees

**Published:** 1878-06-20

**Authors:** 


### NOMINATING COMMITTEES.

The *Medical Bi-weekly* makes the following sensible remarks upon the method of nomination of officers in our medical associations :

"Abolish the nominating committee. It is often the tool of the unworthy. It is seldom the guardian of the most worthy. It can be and has been manipulated, and with this record, it is an unsound and unreliable device. State societies have found it to be so. State societies have, in many instances, abandoned it. The nominations should be made on the floor, and the election should be *viva voce*. The best men only will then be nominated, and one of these will inevitably be elected.

Nominate the really great physicians of this country by open nomination, and elect by the *viva voce* method. This can be done easily and quickly (on the first morning, before tricks and devices are instituted), and the result will give universal satisfaction."

